# Optimization of Cutting Process Parameters in Inclined Drilling of Inconel 718 Using Finite Element Method and Taguchi Analysis

**DOI:** 10.3390/ma13183995

**Published:** 2020-09-09

**Authors:** Salman Pervaiz, Sathish Kannan, Abhishek Subramaniam

**Affiliations:** 1 Department of Mechanical and Industrial Engineering, Rochester Institute of Technology—Dubai Campus, Dubai 341055, UAE; ags3092@rit.edu; 2 Department of Mechanical Engineering, American University of Sharjah, Sharjah 26666, UAE; skannan@aus.edu

**Keywords:** drilling, inconel 718, machining, cutting, finite element method

## Abstract

Nickel-based superalloys are famous in the demanding applications. Inconel 718 is one of the most commonly used nickel-based superalloys due to its extraordinary inherent properties. Inconel 718 is a suitable material for high temperature applications due to the properties such as anti-oxidization, high hot hardness, high creep, and fatigue strength. Drilling operation is one of the most widely used manufacturing operations in almost all industrial sectors. However, drilling operation is very complex in nature due to the presence of intricate geometry of the drill bit. In conventional drilling, cutting is performed by the combined action of the chisel edge and the two or more cutting lips. In depth analysis of the cutting process shows that chisel edge starts with an indentation at the center of the twist drill. Then away from the center, chisel edge performs orthogonal cutting with negative rake angle. Whereas, cutting action at the cutting lip is oblique in nature, and force analysis involves the use of element formulation due to involvement of radius. It is rarely found in the literature where drilling operation at different inclination angles is conducted and analyzed. The presented study numerically investigates the cutting performance of drilling operation, when operated at different inclination angles. The study revealed cutting force variation at different inclination angles due to the different tool workpiece engagement for each inclination. The magnitude of thrust force increased when inclination angle is changed from 30° to 60°. It can be linked with the higher chip load initially in this case as compared to the 30° inclination angle. The cutting temperature was affected by spindle speed (53.7%), followed by feed rate (33.31%) and inclination angle (3.44%).

## 1. Introduction

Nickel-based high performance alloys such as Inconel 718 are favored over other conventional metals and alloys due to its ability to withstand high operating temperatures, high creep, and fatigue resistance. On the other hand, Inconel 718 shows poor machinability performance due to the formation of high cutting forces during machining, elevated cutting temperatures, severe work hardening and significant tool wear. Several researchers have focused their work on investigating the machinability of Inconel 718 using conventional machining processes such as turning, milling, drilling, and grinding processes. D’Addona et al. [1] investigated the machining performance of Inconel 718 under high cutting speeds. The study used surface finish and wear on the cutting tool as preferred output parameters to analyze the cutting performance. The study revealed that tool wear was very rapid at higher level of cutting speed. Shokrani et al. [2] also investigated the machining performance of Inconel 718 using green and novel hybrid cooling strategies. The study investigated the influence of minimum quantity lubrication (MQL), Cryogenic, and CryoMQL strategies for milling of Inconel 718. The study revealed that tool life was doubled. Surface finish was also improved by 18%. Marques et al. [3] investigated the machinability of Inconel 718 under MQL cooling by using ceramic cutting inserts. Ceramic tools were basically whisker reinforced tools. The study also used MoS_2_ solid lubricant in the vegetable oil-based cutting fluid using MQL strategy. This method proved to be an effective option as it reduced the cutting force.

**Figure 1:**
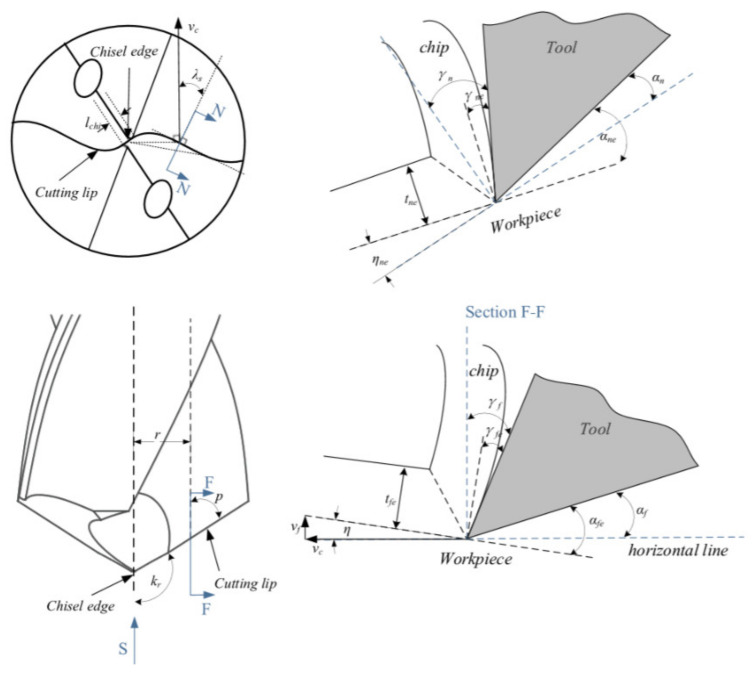
Schematic view of cutting geometry with cross sectional view at different selected elements (with kind permission from Elsevier [11]).

The drilling process involves very complex cutting dynamics due to the involvement of complicated geometry of the cutting edge. A cutting process with the twist drill bit is divided into several portions. Initially chisel edge penetrates in the workpiece and extrusion with plastic deformation happens in the center of the drill bit [4]. Away from the center at the chisel edge, orthogonal cutting takes place with a negative rake angle [5]. However, the cutting lip cuts material under oblique cutting formulation and with increasing radius outward, the machining parameters change too [6,7]. Figure 1 represents the engagement of chisel edge and cutting lip in the conventional drilling process. Yan et al. [8] developed an approach where mechanistic model was presented to analyse the thrust force and torque during the drilling operation of unidirectional CFRP. The study also used a finite element model to figure out the force coefficient for efficient prediction of results. Experimental validation was also performed, and predicted results were found in good agreement with the experimental data. Chatterjee et al. [9] performed a simulation-based study for the drilling operation. The study used difficult to cut titanium alloy in the simulated work. The study performed parametric optimization using forces, torque, entry, and exit circularity of drilled hole as output responses. Attanasio et al. [10] developed a finite element model for the drilling process using Inconel 718 as a workpiece material. The study was focused on figuring out the tool wear involved during the cutting process. The study provided very good agreement with the experimental readings. Schematic illustration of different elements on drill geometry is represented in Figure 1 [11]. Rotary ultrasonic elliptical machining (RUEM) is proved to be an alternative method to reduce delamination in drilling process for CFRP [12]. Cryogenic cooling proved to be more efficient then dry drilling [13].

The Finite Element Method (FEM) is a very beneficial method due to its ability to simulate real-life conditions. Finite Element Analysis (FEA) can be applied to optimize the machining parameters and also to predict results for experiments which have not or could not be carried out. In a study carried out by Nasralla et al. [14], the machining parameters chosen were cutting speed, feed rate, and depth of cut. The levels of cutting speed are 60, 80, and 100 m/min; the levels of feed rate are 0.05, 0.15, and 0.25 mm/rev and levels of depth of cut are 0.2, 0.25, and 0.3 mm. The objective of this study was to minimize the cutting force. Using the Johnson Cook (JC) constitutive model, the simulation was carried out accurately with an average discrepancy of 10% between the simulation and the experiment. The most significant factor in this case was depth of cut (48%) followed by cutting speed (23%) and lastly feed rate (21%). High strength alloys such as Inconel 718 require a lot of energy to be machined. With virtual simulations, the cutting forces at the drill point and the temperature generated can be calculated. Ucak et al. [15] conducted finite element simulations on Inconel 718. The simulation was carried out using an uncoated carbide drill at a cutting speed = 15 m/min, point angle = 140 degrees and feed rate = 0.02 mm/rev. Experimental results indicated a maximum temperature of 500 °C while calculations showed a max. temperature of 453.1 °C. This model can be used to accurately measure temperature at the drill point. Niranjan et al. [16] conducted a study to analyse the effect of parameters such as cutting speed, feed rate, and depth of cut on the turning operation of AA6061 T6 rods which are precipitation hardened. The optimum surface completion and material expulsion rate were determined using contour plots. Pairwise comparisons of the input parameters revealed that a cutting speed more prominent than 400 m/min and a feed rate lower than 0.11 mm/rev produced the best surface completion along with a depth of cut of 1–2 mm. For the same cutting speed, the material removal rate was optimized using a feed rate of 0.015 m/min and a depth of cut of 2 mm. Eltaggaz and Deiab [17] performed a comparative study about conventional and peck drilling of Ti6Al4V. Due to improved chip removal, peck drilling provided better performance.

Machining operations must have a lubricant or a coolant to ease the effect of friction and temperature on the cutting tool and the sample. The effect of different cooling strategies was investigated by Eltaggaz et al. [18]. The research consisted of four levels of cooling techniques, namely dry, flood, minimum quantity lubrication (MQL), and nanofluid. The machining parameters chosen were cutting speed and feed rate with three and two levels, respectively. The trials were conducted at a constant depth of cut of 0.5 mm. It was found that nanofluid cooling technique gives the best surface finish due to factors such as improved wettability of the object surface and a more effective lubrication at the contact point of the tool and sample. Using scanning electron microscopy (SEM), adhesive and abrasive tool wear were noticed in dry and nanofluid cooling techniques. Both flood cooling and MQL showed signs of chipping on the tool inserts. Additionally, in flood cooling, oxidation wear was also noticed. Even though flood cooling offered better results at some places, it was disregarded as the best lubrication technique due to its effect on the environment. Khanafer et al. [19] investigate the performance of Nano-MQL when micro-drilling Inconel 718. The study revealed that Al_2_O_3_ Nanofluids can easily penetrate in the cutting zone and reduce friction using the ball bearing effect.

Guo et al. [20] optimized the energy consumption during the turning process while minimizing surface roughness. A two-step procedure was considered. First, a pre-determined surface roughness value is taken. The input parameters necessary to obtain the required the said roughness value are then determined. After obtaining the factor values, the optimization process begins where the energy consumption was to be minimized. Specific energy which is defined as the ratio of the power consumed to the material removal rate increases with increasing speed and decreases with an increase in feed rate and depth of cut. This study shows that up to 9% energy can be saved by choosing the optimum speed. Zhou et al. [21] estimated the degree to which input parameters play a role in power consumption by using the Genetic Algorithm approach. The conclusion is that increasing the cutting speed decreases energy consumption and processing time. A larger depth of cut also decreases the energy consumption due to a higher Material Removal Rate (MRR). Increasing spindle speed increases energy consumption. It was also seen that decreasing feed rate and the radial cut depth results in energy wastage. Vankanti and Ganta [22] optimized the process parameters in the drilling operation of glass fiber reinforced polymer (GFRP). The controllable parameters chosen were cutting speed, feed rate, point angle, and chisel edge width; and the responses were thrust force, torque, circularity, and surface finish. This work concluded that feed rate was the most influential factor for thrust force while cutting speed affected torque the most. Overall, feed rate and cutting speed were the most significant factors among the four input parameters. Using signal to noise ratios, the optimum parameters for each response were also chosen.

Several researchers have used design of experiments related approaches to optimize different cutting processes. Gopalsamy et al. [23] conducted a study to examine the effects of machining parameters on the machining of hardened steel. The four parameters selected were namely cutting speed, feed, depth of cut and width of cut. The responses selected for the end-milling process were surface finish, tool wear (and tool life (min.). By using the Taguchi method for design of experiments, the multi-response optimization was done by minimizing surface roughness and tool wear and maximizing the tool life. Throughout the process of machining, analysis of the tool wear and the mode of failure of machining tool was done using an optical microscope and SEM. It was noticed in their study that the tool life reduced when the cutting speed exceeded 204 m/min due to an increase in temperature at the point of contact. The material removal rate (MRR) increases when both feed and cutting speed, are high. Cutting speed was considered to be the most influential factor in determining tool life and surface roughness.

**Figure 2:**
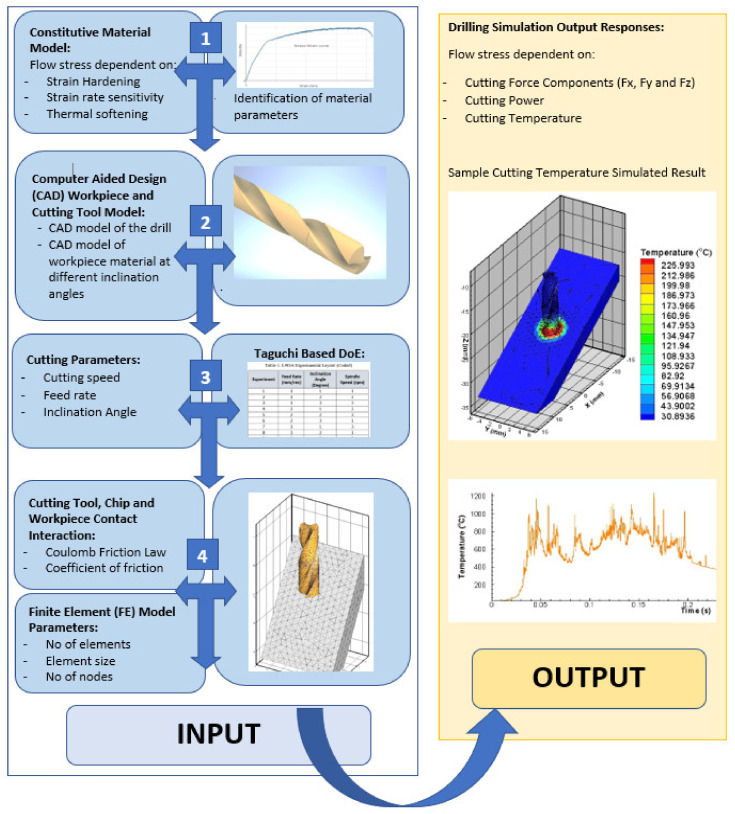
Flow chart of 3D Finite Element (FE) drilling model at various inclination angles.

It is rarely found in the literature where drilling operation at different inclination angles is conducted and analyzed. In this paper, an effort has been made to numerically investigate the machining performance of drilling Inconel 718 at various inclination angles. The study revealed interaction of intricate twist drill’s cutting-edge geometry when inclination angles are incorporated. The study revealed simulated signatures of cutting forces, power, and temperature as output responses. The output responses were analyzed further using famous Taguchi’s design of experiments-based approach to draw reasonable conclusions. For better understanding of the viewers, Figure 2 shows the flow chart of steps performed toward the inputs and output responses of this study. Table 1 represents nomenclature.

## 2. Three-Dimensional (3D) Finite Element (FE) Drilling Simulation Setup

It is important to generate the 3D drilling finite element model with appropriate details to capture the complex output response. In other to do so, NX CAD Siemens software package (Simens PLM Software, Plano, TX, USA) was used for computer aided design (CAD) models of twist drill and workpiece geometry. CAD models were then transferred into the finite element package namely Third Wave System’s AdvantEdge, Minneapolis, MN, USA. The software works on the explicit Lagrangian formulation. Software provides coupled thermo-mechanical transient analysis that is required for the machining simulations.

The workpiece material of Inconel 718 was selected from the AdvantEdge’s library because material modeling is not the scope of this study. The software by default uses power law to describe workpiece materials behavior. The power law applied in this study is described in Equation (1). The power law includes the influence of strain hardening function as g (ε*^p^*), thermal softening function as θ(T) and rate sensitivity function as δ (έ).

(1) $$\sigma \left(\varepsilon^{p},T,\dot{\varepsilon }\right)=g\left(\varepsilon^{p}\right)\theta \left(T\right)\delta \left(\dot{\varepsilon }\right)$$

In the material model, strain hardening function is governed by Equation (2). Strain hardening is evaluated using the stress strain data on material. Strain rate sensitivity function is governed by Equation (3). Thermal softening function is controlled in the power law with fifth order polynomial equation as depicted in Equation (4).

(2) $$g\left(\varepsilon^{p}\right)=\sigma o{\left[1+\frac{\varepsilon^{p}}{\varepsilon^{p}o}\right]}^{\frac{1}{n}}\;if\;\varepsilon^{p}<\varepsilon_{cut}^{p}$$

In Equation (2), σo is the initial yield stress, $\varepsilon^{p}$ is plastic strain, $\varepsilon_{o}^{p}$ is reference plastic strain, 1/n is strain hardening power. The initial yield stress is found by using stress strain from uniaxial compression or tensile test of workpiece material. The values of $\varepsilon_{o}^{p}$ and n is obtained by curve fitting the stress strain data.

(3) $$\delta \left(\dot{\varepsilon }\right)=\sigma o{\left[1+\frac{\dot{\varepsilon }}{\dot{\varepsilon }o}\right]}^{\frac{1}{m1}}$$

In the Equation (3), the function represents the flow stress behavior at higher strain rates. In this equation $\dot{\varepsilon }$ is plastic strain rate, and ${\dot{\varepsilon }}_{o}$ is reference plastic strain rate and m1 is strain rate sensitivity.

θ(T) = c_0_ + c_1_T^1^ + c_2_T^2^ + c_3_T^3^ + c_4_T^4^ + c_5_T^5^ if T < T_cut_ (4)

(5) $$\theta \left(T\right)=\theta \left(T_{cut}\right)\left(1-\frac{T-T_{cut}}{T_{m}-T_{cut}}\right)\;if\;T>T_{cut}$$

In Equations (4) and (5), thermal softening function is represented. Thermal softening function is composed of 5th order polynomial with c_1_–c_5_ constants. These constants c_1_–c_5_ are curve fitted by the compression test data at elevated temperatures. Where T is the temperature during the test. Equation (6) shows the governing Coulomb’s friction law used in this simulation.

(6) $$\tau =\mu \sigma_{n}$$

**Figure 3:**
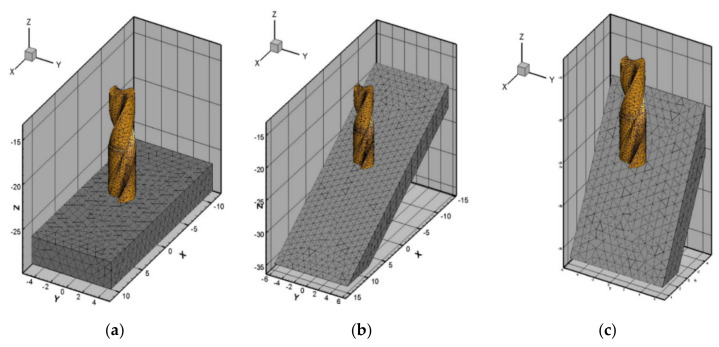
3D Finite Element (FE) drilling model at various inclination angles (**a**) 90° Inclination (**b**) 60° Inclination, (**c**) 30° Inclination.

In Thirdwave AdvantEdge package damage is controlled through a damage function. The damage function D is mentioned in Equation (7) as under [24].

(7) $$D=\underset{i}{\sum }\frac{\Delta \varepsilon_{i}^{p}}{\varepsilon_{f_{i}}^{p}}$$

where D is cumulative damage, $\Delta \varepsilon_{i}^{p}$ is instantaneous increment of strain and $\varepsilon_{f_{i}}^{p}$ is instantaneous strain to failure. Figure 3 represents the finite element model developed for drilling at different inclination angles. The volume mesh (tool and workpiece) consists of four nodes tetrahedral elements. The manual and available literature [25] were used in order to set the default meshing parameters. For cutting tool, maximum element size and minimum element size was selected to be 0.3 mm and 0.03 mm respectively. To decide the nature of mesh transition, mesh grading factor was selected as 0.4. To control mesh accuracy, curvature safety was selected to be 1.5. Density of elements on any edge was selected to be 0.5 using segment per edge parameter. For the workpiece, maximum element size and minimum element size was selected as 3 mm and 0.1 mm respectively.

### Experimental Validation of Material Model Using Orthogonal Cutting

**Figure 4:**
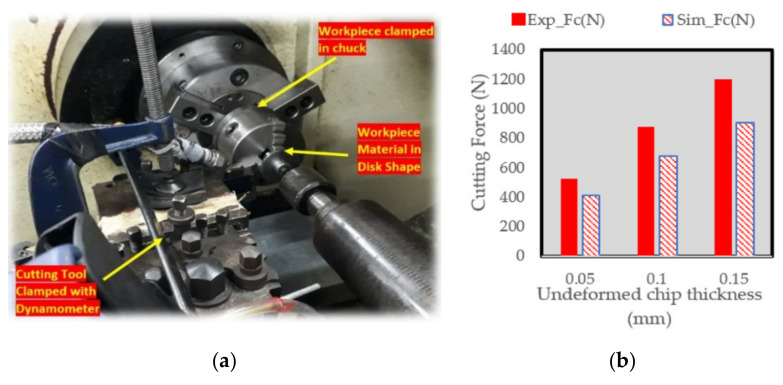
(**a**) Orthogonal cutting validation setup (**b**) Experimental and Simulated Cutting Forces at different values of undeformed chip thickness.

To validate the material model for this work, orthogonal cutting validation setup was used as shown in Figure 4. The computer numeric control (CNC) turning center was model Excel BNC-21437 and the controller of the machine was the Fagor 800T. The cutting forces were examined using the Kistler multi-channel dynamometer with the product code number 9129 AA. The error between the experimental and simulated forces was found to be in the range of 10–20%.

## 3. Experimental Design

The Taguchi method is a statistical approach to improve and optimize experimental parameters. As a Design of Experiment (DOE) technique, it is used to develop an efficient design to conduct the experiment. Developed in the 1970s and 1980s, this method quickly became popular as it could be used to improve the quality of manufactured products in several industries such as agriculture, manufacturing, medicine, and engineering. Using this technique, one can reduce costs by effectively minimizing the number of runs required to examine the effect of each factor. In this work, we studied the effect of three factors, namely feed rate, inclination angle and spindle speed on six response parameters (Fx, Fy, Fz, Power, and Temperature). Each of the factors has three levels. Based on the input parameters, the Taguchi L9 Orthogonal Array was chosen. Selection of cutting parameters was performed logically in consultation with the available literature for Inconel 718. Accordingly, data collection was carried out using simulation (Table 2). To optimize the parameters, the criteria of optimization were first established. As cutting forces and torque reflect the effort required for cutting, the study minimized it to maximize the performance. The power (and energy consumption) was minimized as well to reduce the costs. The temperature at the contact points can increase drastically due to friction. The usage of Inconel in our case meant that the temperature at the contact points were very high (~450–800 °C). Thus, this response was minimized as well to reduce the possibility of extreme tool wear and breakage. An indirect consequence of our efforts is also maximizing tool life. Table 3 shows the cutting parameters along with their levels. Signal-to-Noise (S/N) ratios were calculated to determine the quality characteristic. A signal is a desirable value for the output while noise is an undesirable value for the output. Therefore, by maximizing the S/N ratio, we maximize the quality. Depending on the target value, different S/N ratios were calculated. Here smaller is better; the criterion as shown in Equation (8) was adopted.

(8) $$S/N\;ratio\;=-10\;log\left(\frac{1}{n}\Sigma y_{i}^{2}\right)$$

Analysis of variance (ANOVA) is a method to understand the significance of an input parameter on the overall response. By comparing the means of output for different input parameters, it can be used to determine which input parameters are statistically different. The Taguchi method alone cannot be used to show the significance of a factor. When it is used alongside ANOVA, the significance can be quantified. The main components of an ANOVA table are the sum of squares (SS), degrees of freedom (DF), F-value and *P*-value. The sum of squares was calculated using:

(9) $$SS_{total}=\overset{n}{\underset{i=1}{\sum }}\left(\eta_{i}-\bar{\eta }\right)$$

(10) $$SS_{factor}=\overset{n}{\underset{i=1}{\sum }}\left(\eta_{j}-\bar{\eta }\right)$$

where n is the number of runs or experiments, η_i_ is the S/N ratio of the response at the i^th^ experiment, η_j_ is the S/N ratio of the response for the j^th^ factor and $\bar{\eta }$ is the mean of S/N ratios of the response for all experiments. 

Degrees of freedom for a factor with n levels is n − 1. The Adjusted *MS* for a factor can be calculated using:

(11) $$Adj\;MS=\frac{SS}{DF}$$

The F-value was calculated using:

(12) $$\mathrm{F-}value=\frac{Adj\;MS}{{Adj\;MS}_{total}}$$

The contribution of each factor was calculated using:

(13) $$\%Contribution=\frac{{SS}_{factor}}{{SS}_{total}}\times 100$$

The *P*-value was defined for a confidence level of 95%. The F-test was also used. The critical F-value is usually around 5.

## 4. Results and Discussion

### 4.1. Analysis of Cutting Forces

**Figure 5:**
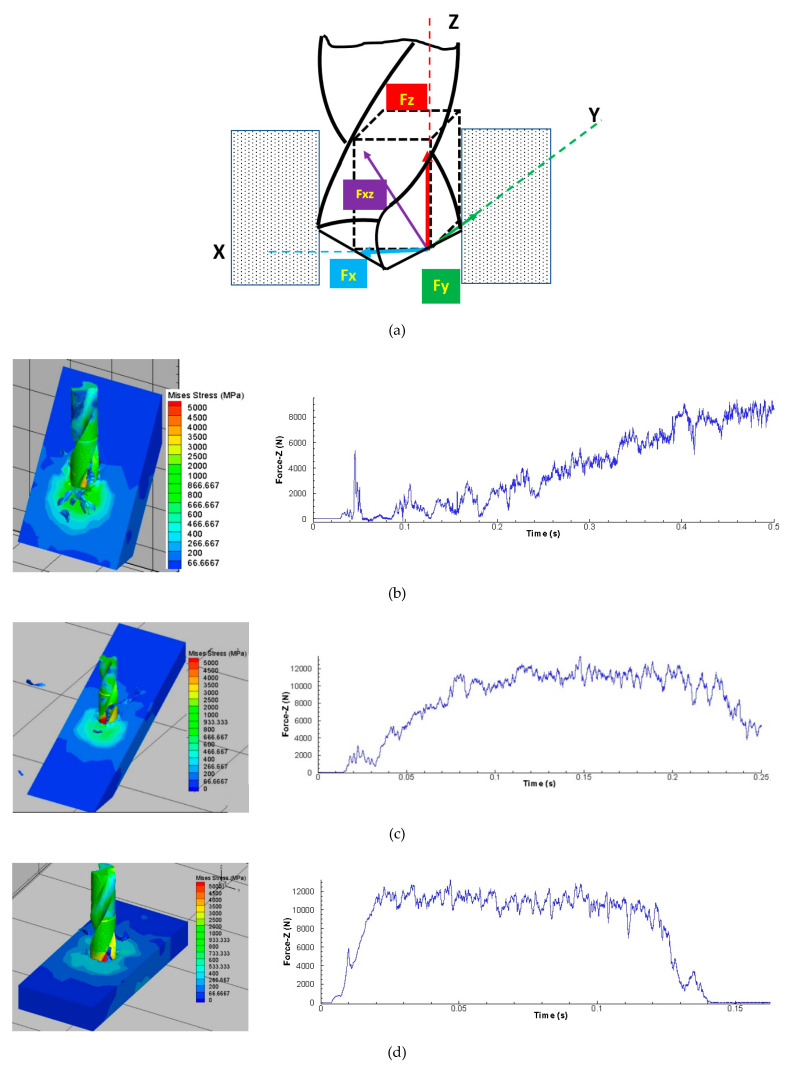
(**a**) Cutting force components (kind permission from Elsevier [26]) (**b**) FE Simulation of 30° inclination angle (0.5 mm/rev, 1000 spindle rpm) (**c**) FE Simulation of 60° inclination angle (0.5 mm/rev, 2000 spindle rpm) (**d**) FE Simulation of 90° inclination angle (0.5 mm/rev, 3000 spindle rpm).

Cutting forces are regarded as one of the most important machining performance indicators. Technically speaking, the cutting force represents shearing action of a workpiece material when cutting edge is in contact, and also depicts the amount of power consumed and energy required to cut the desired material and geometry. In the drilling operation, cutting forces are generated a bit differently from the combined cutting action of chisel edge and the cutting lip. A generic representation of cutting force components has been reported in Figure 5a [26]. Where the force is decomposed into components of tangential force (Fy) and force (Fxz) that is perpendicular to the cutting edge in this case. This force component (Fxz) is further divided into two components namely radial force (Fx) and thrust force (Fz) respectively. Keeping in view that there are two cutting lips, total thrust force is computed by taking into account the thrust forces generated by both of the lips. It is also worth noticing that the two radial force components are balancing each other. The cutting simulated thrust force signal has been shown for different inclination angles. It can be easily seen that at 30° inclination angle, the chisel edge indented the material and smoothly enters the material with the one-sided cutting lip.

The magnitude of thrust force is a getting higher when the inclination angle is changed from 30° to 60°. It can be linked with the higher chip load initially in this case as compared to the 30° inclination angle. Inclination of 90° provided the traditional signature, where initial ramp in the thrust force is linked with the chisel edge cutting at the entry of drill bit into the material. The middle region represents engagement of cutting lips with workpiece material. At the end, drop in the thrust force represents exit of the cutting bit. Table 4 shows the responses for the Taguchi L9 orthogonal array.

Table 5, Table 6 and Table 7 show the Taguchi analysis of the cutting forces where data is converted into the S/N ratios. As described previously the quality characteristic used for cutting forces was “smaller the better”.

Table 5 shows the ANOVA conducted on cutting forces. For cutting force in x-direction (Fx), feed rate had the highest contribution (92.95%) followed by spindle speed (5.26%) and inclination angle (1.48%). It can be observed that the feed rate affects Fx and Fy the most out of the three input parameters while spindle speed affects Fz the most. Comparing the F-value of each factor against the critical F-value, it can be concluded that feed rate and spindle speed are significant in affecting Fx. In the y-direction, cutting force (Fy) is affected mainly by feed rate which has a percent contribution of 84.55, followed by inclination angle (3.5%) and then spindle speed (3.34%). Using *p*-value and F-value comparisons, only feed rate was seen as the significant factor while inclination angle and spindle speed were insignificant. Cutting force in z-direction (Fz) was influenced by spindle speed with a contribution of 43.16%, followed by feed rate (35.49%) and inclination angle (16.65%).

**Figure 6:**
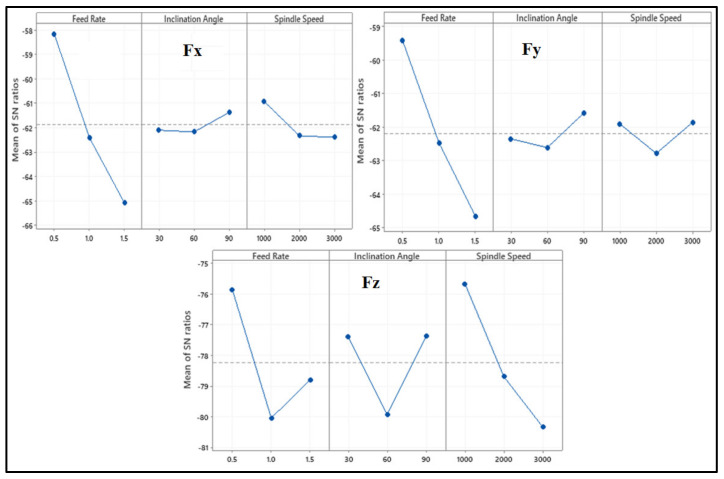
Main Effects Plot of S/N ratios for Fx, Fy and Fz.

Table 7 was constructed using the S/N ratios of cutting force components using the average value of the experiments for the levels 1,2 and 3. Similarly the S/N ratios of other parameters was also investigated and reported in the table. The optimum level of the controlling factor is reported in Figure 6. It can be seen in Figure 6 that the optimal setting for Fx is feed rate of 0.5 mm/rev, inclination angle of 90° and spindle speed of 1000 rpm. A feed rate of 0.5 mm/rev, inclination angle of 90° and spindle speed of 3000 rpm was seen to minimize the cutting force in the y-direction. Since the difference in the effect of the spindle speed of 1000 rpm and 3000 rpm was so small on Fy, it can be said that Fx and Fy show similar trends with regards to the optimal setting of input parameters. For the cutting force in the z-direction, the setting with feed rate of 0.5 mm/rev, inclination angle of 90° and spindle speed of 1000 rpm was optimal.

### 4.2. Analysis of Cutting Power

**Figure 7:**
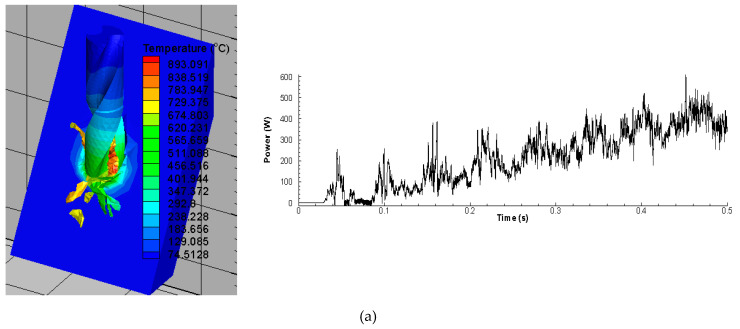

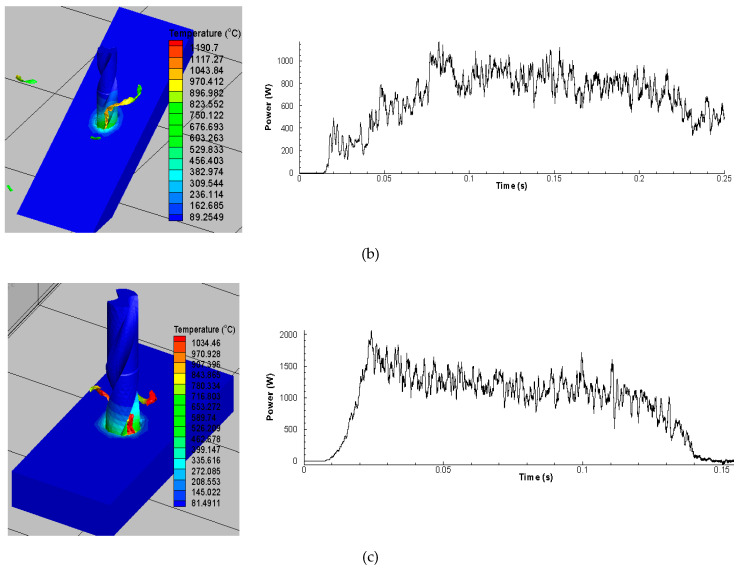
Power signal captured (**a**) FE Simulation of 30° inclination angle (0.5 mm/rev, 1000 spindle rpm) (**b**) FE Simulation of 60° inclination angle (0.5 mm/rev, 2000 spindle rpm) (**c**) FE Simulation of 90° inclination angle (0.5 mm/rev, 3000 spindle rpm).

Power consumed in the cutting process is an important indicator of machining performance. It is vital element to analyse the overall energy consumed in the cutting process when analyzed with respect to time. It is mainly estimated by using the product of main cutting force component with the cutting speed (P = FV). Figure 7 shows traditional power signatures attained using the drilling simulations. To compute further these power signals, average values were calculated and used further in Taguchi analysis.

**Figure 8:**
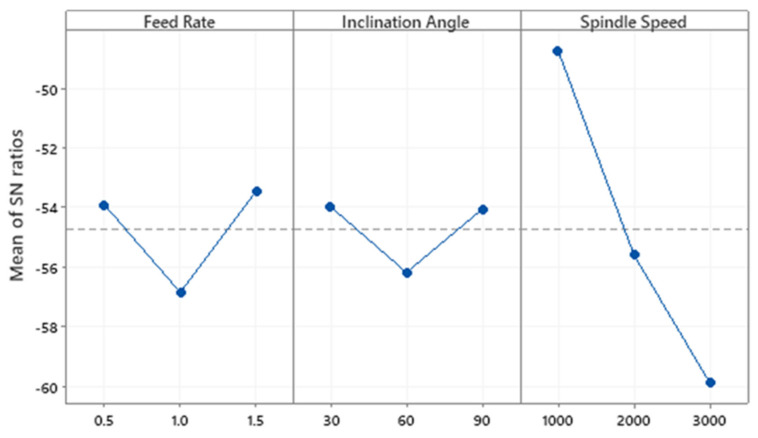
Main effects plot of S/N ratios for power.

Table 8 represents the power responses and their respective S/N ratios. Table 9 shows ANOVA conducted on Power. Spindle speed has the highest contribution to power (85.58%), followed by feed rate (9.22%) and inclination angle (4.22%). The F-test indicates that the feed rate and spindle speed are significant while inclination angle is not. This could be due to the fact that power increases with spindle speed. Table 10 shows the Taguchi method analysis of Power data using Signal-to-Noise ratios. This response parameter was minimized. It has been constructed using the S/N ratios of cutting force components using the average value of the experiments for the levels 1, 2, and 3. The optimal setting for power for maximizing S/N ratios is feed rate of 1.5 mm/rev, inclination angle of 30° and spindle speed of 1000 rpm as shown in Figure 8.

### 4.3. Analysis of Cutting Temperature

Cutting temperature is also considered one of the significant parameters to examine the machining performance of cutting processes. Cutting temperature should be minimized to enhance the over-all performance. One major reason is the tool wear that is highly dependent on the cutting temperature. Cutting temperature has tendency to active and control cutting tool wear mechanisms such as adhesion, diffusion, and abrasion etc. Several tool failure modes such as excessive plastic deformation, attrition, crater formation, built-up-edge formation, thermal cracks, and tool edge softening can also be linked with the cutting temperature. Due to all these negative effects coolants or lubricant are used in the cutting operation to dissipate heat appropriately. Table 11 represents the cutting temperature responses and their respective S/N ratios. Table 12 shows ANOVA conducted on cutting temperature. Table 12 shows the ANOVA conducted on Temperature. Temperature was affected by spindle speed (53.7%), followed by feed rate (33.31%) and inclination angle (3.44%).

**Figure 9:**
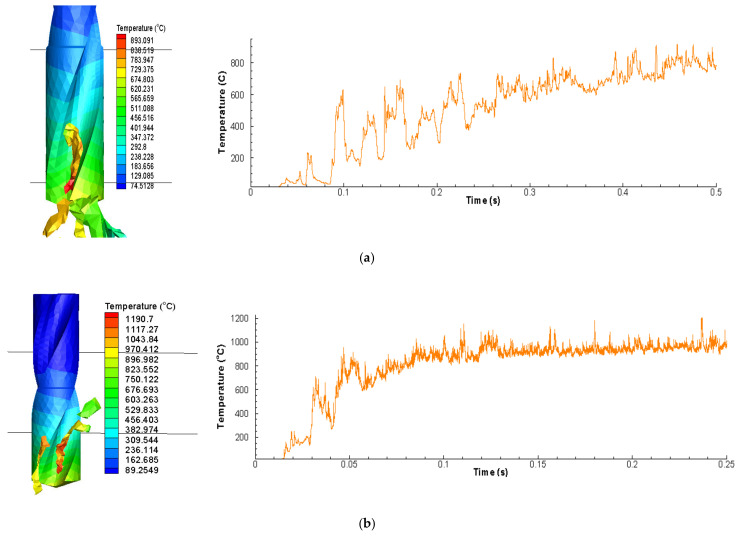

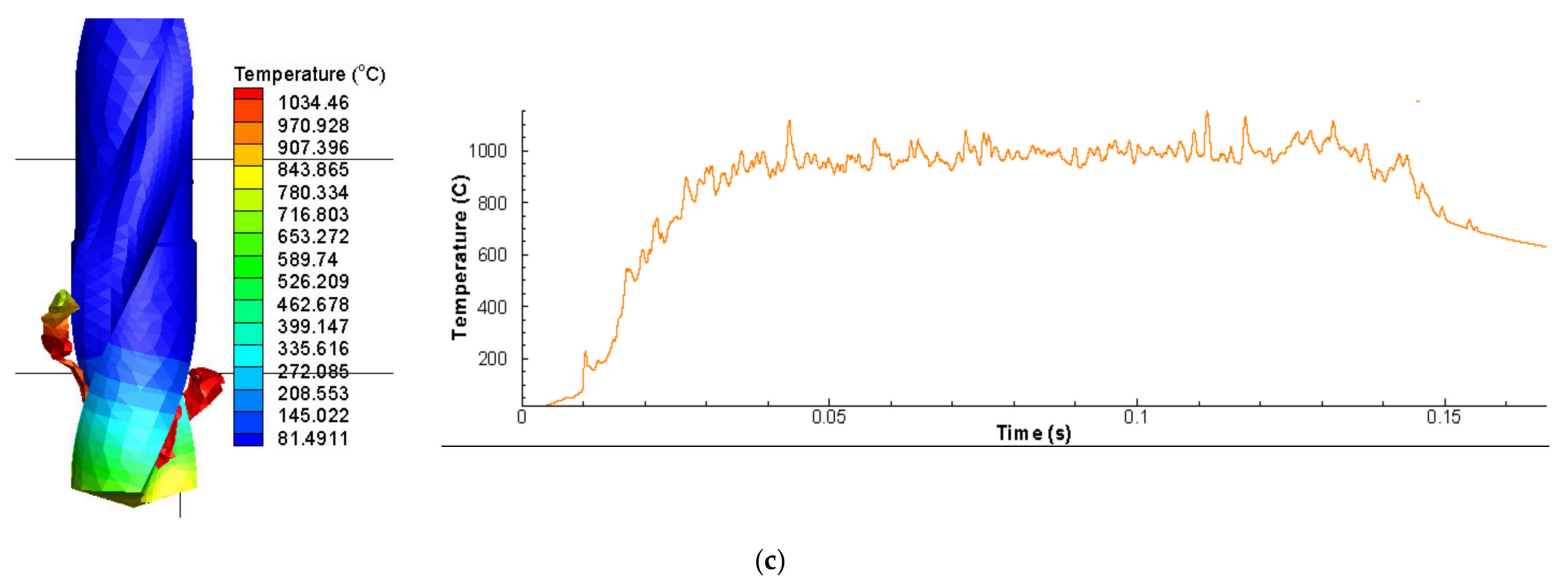
Cutting temperature captured (**a**) FE Simulation of 30° inclination angle (0.5 mm/rev, 1000 spindle rpm) (**b**) FE Simulation of 60° inclination angle (0.5 mm/rev, 2000 spindle rpm) (**c**) FE Simulation of 90° inclination angle (0.5 mm/rev, 3000 spindle rpm).

**Figure 10:**
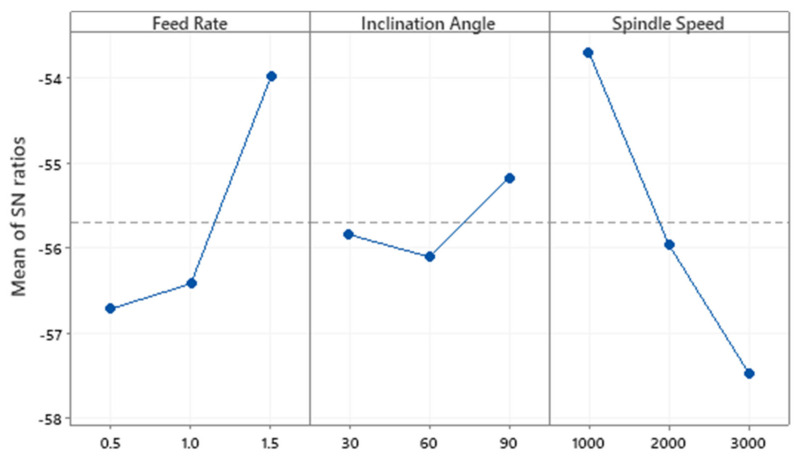
Main Effects plot of S/N ratios for Temperature.

Figure 9 shows the cutting temperature signature obtained using different conditions and inclination angle. Table 11 and Table 12 show the Taguchi analysis of cutting temperature data. Table 12 shows the ANOVA conducted on cutting temperature. Cutting temperature was affected by spindle speed (53.7%), followed by feed rate (33.31%) and inclination angle (3.44%). The objective was to minimize cutting temperature. Based on Table 13, the main effects plot for S/N ratios was plotted in Figure 10. The main effects revealed that the best setting for minimizing temperature is feed rate of 1.5 mm/rev, inclination angle of 90° and spindle speed of 1000 rpm.

## 5. Conclusions

The study simulated the concept of inclined drilling in the light of cutting force components, cutting power, and cutting temperature. The following conclusions are drawn from this investigation:

1. In 30° inclination angle, the thrust force has a ramping signature throughout the cutting process that represents the ramping increase in chip load starting from the entry of drill. Also represents engagement of chisel and cutting edge with the workpiece material.
2. The magnitude of thrust force increased when inclination angle is changed from 30° to 60°. It can be linked with the higher chip load initially in this case as compared to the 30° inclination angle.
3. For cutting force component in x-direction (Fx), feed rate found to be more dominant and has the highest contribution (92.95%) followed by spindle speed (5.26%) and inclination angle (1.48%). In the y-direction, cutting force (Fy) is affected mainly by feed rate which has a percent contribution of 84.55, followed by inclination angle (3.5%) and then spindle speed (3.34%). It can be observed that the feed rate affects Fx and Fy the most out of the three input parameters while spindle speed affects Fz the most.
4. For lower thrust cutting force, the setting with low feed rate of 0.5 mm/rev, inclination angle of 90° and low spindle speed of 1000 rpm was optimal.
5. The spindle speed has the highest contribution to power (85.58%), followed by feed rate (9.22%) and inclination angle (4.22%).
6. The cutting temperature was affected by spindle speed (53.7%), followed by feed rate (33.31%) and inclination angle (3.44%).
7. The study will be beneficial for different industrial sectors as more output performance parameters such as tool wear and energy consumption can be predicted using the force, temperature, and power data available in this work.

## Figures and Tables

**Table 1:** Table of Nomenclature.

Nomenclature			
A	Yield stress (Ref. condition)	MQL	Minimum quantity lubrication
B	Strain hardening constant	DoC	Depth of cut
n	Strain hardening coefficient	Vc	Cutting speed
C	Strengthening coefficient—strain rate	RPM	Spindle speed
m	Thermal softening coefficient	fz	Feed rate
µ	Friction coefficient	σ	Equivalent stress
D	Damage parameter	ε	Equivalent plastic strain

**Table 2:** Factors and their associated levels.

Factors	Symbol	Levels (Coded)	Levels (Uncoded)
Feed Rate (mm/rev)	A	1 2 3	0.5 1 1.5
Inclination Angle (Degree)	B	1 2 3	30 60 90
Spindle Speed (rpm)	C	1 2 3	1000 2000 3000

**Table 3:** Orthogonal Array L9 (3 × 3) experimental Layout (Coded).

Experiment	A: Feed Rate (mm/rev)	B: Inclination Angle (Degree)	C: Spindle Speed (rpm)
1	1	1	1
2	1	2	2
3	1	3	3
4	2	1	2
5	2	2	3
6	2	3	1
7	3	1	3
8	3	2	1
9	3	3	2

**Table 4:** Taguchi’s L9 orthogonal array responses of cutting forces, power, and temperature.

Exp. No.	Feed Rate (mm/ rev)	Inclination Angle (Degree)	Spindle Speed (rpm)	Fx (N)	Fy (N)	Fz (N)	Power (W)	Temperature (°C)
1	0.5	30	1000	743.22	1029.81	3786.83	211.34	495.99
2	0.5	60	2000	860.08	974.40	8314.40	652.61	776.29
3	0.5	90	3000	827.47	818.87	7614.17	885.55	834.76
4	1	30	2000	1457.63	1412.59	10,220.23	750.82	732.83
5	1	60	3000	1448.24	1498.41	14,032.19	1383.23	763.28
6	1	90	1000	1088.13	1110.85	7078.90	325.29	519.06
7	1.5	30	3000	1908.80	1550.52	10,505.76	786.87	654.49
8	1.5	60	1000	1702.09	1692.23	8367.29	296.48	439.51
9	1.5	90	2000	1786.71	1904.36	7499.99	446.26	435.51

**Table 5:** The measured cutting force responses and the corresponding signal to noise ratios.

Feed Rate (mm/rev)	Inclination Angle (Degree)	Spindle Speed (rpm)	Fx (N)	Fy (N)	Fz (N)	S/N Ratio for Fx (dB)	S/N Ratio for Fy (dB)	S/N Ratio for Fz (dB)
0.5	30	1000	743.22	1029.81	3786.83	−57.4223	−60.2552	−71.5655
0.5	60	2000	860.08	974.40	8314.40	−58.6908	−59.7748	−78.3966
0.5	90	3000	827.47	818.87	7614.17	−58.3551	−58.2643	−77.6325
1	30	2000	1457.63	1412.59	10,220.23	−63.2729	−63.0003	−80.1892
1	60	3000	1448.24	1498.41	14,032.19	−63.2168	−63.5126	−82.9425
1	90	1000	1088.13	1110.85	7078.90	−60.7336	−60.9131	−76.9993
1.5	30	3000	1908.80	1550.52	10,505.76	−65.6152	−63.8096	−80.4285
1.5	60	1000	1702.09	1692.23	8367.29	−64.6196	−64.5692	−78.4517
1.5	90	2000	1786.71	1904.36	7499.99	−65.0411	−65.595	−77.5012

**Table 6:** Results of the ANOVA for Cutting force.

Response	Source	DF	Seq SS	Contribution (%)	Adj SS	Adj MS	F-Value	*p*-Value
Fx	Feed Rate	2.00	73.3889	92.95	73.39	36.6944	301.36	0.003
	Inclination Angle	2.00	1.1722	1.48	1.17	0.5861	4.81	0.172
	Spindle Speed	2.00	4.1535	5.26	4.15	2.0767	17.06	0.055
	Error	2.00	0.2435	0.31	0.24	0.1218		
	Total	8.00	78.9581	100.00				
Fy	Feed Rate	2	41.346	84.55	41.346	20.6728	9.82	0.092
	Inclination Angle	2	1.71	3.50	1.71	0.8552	0.41	0.711
	Spindle Speed	2	1.634	3.34	1.634	0.8168	0.39	0.721
	Error	2	4.212	8.61	4.212	2.1062		
	Total	8	48.902	100.00				
Fz	Feed Rate	2	27.603	35.49	27.603	13.802	7.54	0.117
	Inclination Angle	2	12.947	16.65	12.947	6.473	3.54	0.22
	Spindle Speed	2	33.565	43.16	33.565	16.782	9.17	0.098
	Error	2	3.66	4.71	3.66	1.83		
	Total	8	77.775	100.00				

**Table 7:** Response table for mean S/N ratios for Fx, Fy and Fz.

	Level	Feed Rate (dB)	Inclination Angle (dB)	Spindle Speed (dB)
**Fx**	1	−58.16	−62.10	−60.93
	2	−62.41	−62.18	−62.33
	3	−65.09	−61.38	−62.40
	Delta	6.94	0.80	1.47
	Rank	1	3	2
**Fy**	1	−59.43	−62.36	−61.91
	2	−62.48	−62.62	−62.79
	3	−64.66	−61.59	−61.86
	Delta	5.23	1.03	0.93
	Rank	1	2	3
**Fz**	1	−75.86	−77.39	−75.67
	2	−80.04	−79.93	−78.70
	3	−78.79	−77.38	−80.33
	Delta	4.18	2.55	4.66
	Rank	2	3	1

**Table 8:** The measured cutting power responses and the corresponding signal to noise ratios.

Feed Rate (mm/rev)	Inclination Angle (Degree)	Spindle Speed (rpm)	Power (W)	S/N Ratio for Power (dB)
0.5	30	1000	211.34	−46.4997
0.5	60	2000	652.61	−56.2931
0.5	90	3000	885.55	−58.9442
1	30	2000	750.82	−57.5107
1	60	3000	1383.23	−62.8179
1	90	1000	325.29	−50.2453
1.5	30	3000	786.87	−57.9181
1.5	60	1000	296.48	−49.4398
1.5	90	2000	446.26	−52.9918

**Table 9:** Results of the ANOVA for Power.

Source	DF	Seq SS	Contribution (%)	Adj SS	Adj MS	F-Value	*p*-Value
Feed Rate	2	20.505	9.22%	20.505	10.253	9.45	0.096
Inclination Angle	2	9.388	4.22%	9.388	4.694	4.33	0.188
Spindle Speed	2	190.307	85.58%	190.307	95.154	87.69	0.011
Error	2	2.170	0.98%	2.170	1.085		
Total	8	222.371	100.00%				

**Table 10:** Response table for mean S/N ratios for cutting power.

Level	Feed Rate (dB)	Inclination Angle (dB)	Spindle Speed (dB)
1	−53.91	−53.98	−48.73
2	−56.86	−56.18	−55.60
3	−53.45	−54.06	−59.89
Delta	3.41	2.21	11.17
Rank	2	3	1

**Table 11:** The measured cutting temperature responses and the corresponding signal to noise ratios.

Feed Rate (mm/rev)	Inclination Angle (deg)	Spindle Speed (rpm)	Temperature (°C)	S/N Ratio for Temperature (dB)
0.5	30	1000	495.9862	−53.9094
0.5	60	2000	776.2863	−57.8004
0.5	90	3000	834.7598	−58.4312
1	30	2000	732.8310	−57.3001
1	60	3000	763.2845	−57.6537
1	90	1000	519.0556	−54.3043
1.5	30	3000	654.4875	−56.3180
1.5	60	1000	439.5133	−52.8594
1.5	90	2000	435.5083	52.7799

**Table 12:** Results of the ANOVA for Temperature.

Source	DF	Seq SS	Contribution	Adj SS	Adj MS	F-Value	*p*-Value
Feed Rate	2	13.450	33.31%	13.450	6.7251	3.49	0.223
Inclination Angle	2	1.388	3.44%	1.388	0.6942	0.36	0.735
Spindle Speed	2	21.684	53.70%	21.684	10.8422	5.62	0.151
Error	2	3.857	9.55%	3.857	1.9286		
Total	8	40.380	100.00%				

**Table 13:** Response Table for S/N ratios for Temperature.

Level	Feed Rate (dB)	Inclination Angle (dB)	Spindle Speed (dB)
1	−56.71	−55.84	−53.69
2	−56.42	−56.10	−55.96
3	−53.99	−55.17	−57.47
Delta	2.73	0.93	3.78
Rank	2	3	1
